# Dr. (?) Hunter’s Dental Anodyne

**Published:** 1891-02

**Authors:** 


### DR. (?) HUNTER'S DENTAL ANODYNE.

UNDER "Current News" will be found a copy of a portion of "Specifications forming part of Letters Patent No. 394,693, dated December 18, 1888."

The dangerous character of this concoction requires that it should be exposed promptly. It is only necessary, we hope, to call attention to the facts connected with it to stamp its character. The arsenious acid contained in this preparation is sufficient to kill ten individuals, and that it is certain to destroy all the pulps to which it may be applied is fully understood.

The circular accompanying this remarkable production, after giving an account of its wonderful and "potent ingredients," says: "I have sold one-third interest in my patent right to a Norfolk gentleman, and am now manufacturing my preparation at No. 46 Cumberland Street, Norfolk, Va." He kindly says, "Mothers should not be without it, as they can stop the little ones' teeth from aching in a few minutes."

It is scarcely credible, in view of these facts, that any one should be willing to endorse this compound, yet we are informed that a very prominent dentist of New York City has recommended it. And the inventor (?) says, "I demonstrated my preparation in the Dental College of Philadelphia to the entire satisfaction of professors and students, and they pronounce it a wonderful thing." This, it is hoped, may be proved an incorrect statement.

It is high time the dental profession had put the stamp of ignominy upon all secret and proprietary preparations of whatever kind.

